# EGFR Inhibition Blocks Palmitic Acid-induced inflammation in cardiomyocytes and Prevents Hyperlipidemia-induced Cardiac Injury in Mice

**DOI:** 10.1038/srep24580

**Published:** 2016-04-18

**Authors:** Weixin Li, Qilu Fang, Peng Zhong, Lingfeng Chen, Lintao Wang, Yali Zhang, Jun Wang, Xiaokun Li, Yi Wang, Jingying Wang, Guang Liang

**Affiliations:** 1 Chemical Biology Research Center, School of Pharmaceutical Science, Wenzhou Medical University, Wenzhou, Zhejiang, China; 2 Department of Cardiology, Wenzhou Central Hospital, Wenzhou, 325300, Zhejiang, China

## Abstract

Obesity is often associated with increased risk of cardiovascular diseases. Previous studies suggest that epidermal growth factor receptor (EGFR) antagonism may be effective for the treatment of angiotensin II-induced cardiac hypertrophy and diabetic cardiomyopathy. This study was performed to demonstrate if EGFR plays a role in the pathogenesis of hyperlipidemia/obesity-related cardiac injuries. The *in vivo* studies using both wild type (WT) and apolipoprotein E (ApoE) knockout mice fed with high fat diet (HFD) showed the beneficial effects of small-molecule EGFR inhibitors, AG1478 and 542, against obesity-induced myocardial injury. Administration of AG1478 and 542 significantly reduced myocardial inflammation, fibrosis, apoptosis, and dysfunction in both two obese mouse models. *In vitro*, EGFR signaling was blocked by either siRNA silencing or small-molecule EGFR inhibitors in palmitic acid (PA)-stimulated cardiomyocytes. EGFR inhibition attenuated PA-induced inflammatory response and apoptosis in H9C2 cells. Furthermore, we found that PA-induced EGFR activation was mediated by the upstream TLR4 and c-Src. This study has confirmed the detrimental effect of EGFR activation in the pathogenesis of obesity-induced cardiac inflammatory injuries in experimental mice, and has demonstrated the TLR4/c-Src-mediated mechanisms for PA-induced EGFR activation. Our data suggest that EGFR may be a therapeutic target for obesity-related cardiovascular diseases.

Obesity has become more prevalence nowadays due to the consumption of high lipid or high-energy diet^1^. Visceral fat obesity is often associated with increased risk for diseases such as atherosclerosis, dyslipidemia, hypertension, cerebrovascular thrombosis, diabetes and ischemic heart disease^2^,^3^,^4^. Particularly in the heart, obesity has a strong impact on left ventricle hypertrophy, myocardial fibrosis and atrial fibrillation that eventually leads to heart failure^5^. The underlying mechanism of obesity induced cardiac remodeling with structural and functional abnormalities remain incompletely understood, but with an orchestra of inflammatory, hemodynamic and neurohumoral factors as well as oxidative stress and adipocyte paracrine effect^6^. Therefore, identification of key signaling pathways that control myocardial cell hypertrophy, apoptosis and interstitial fibrosis will provide a better understanding of these pathophysiological changes and may further lead to new opportunities for disease intervention.

Epidermal growth factor receptor (EGFR), also named ErbB1 receptor, is a receptor tyrosine kinase that is expressed in various tissues and mediates cell proliferation, differentiation, migration and survival^7^. EGFR has been well demonstrated as a therapeutic target for cancer treatment. Recently, EGFR has been found to be involved in non-malignant chronic diseases^8^. In cardiovascular system, EGFR and its ligands play a sophisticated role in the regulation of multiple cellular behaviors^9^. During early embryogenesis, EGFR signaling pathway is essential for cardiac growth and development^10^,^11^. The expression of EGFR and its ligands are increased in human vascular lesions of atherosclerosis^12^, even though the impact of EGFR signaling on atherogenesis is not known yet. EGFR transactivation mediates cardiac hypertrophy induced by the overstimulation of angiotensin II type 1 receptor (AT1R)^13^, and antisense to EGFR prevents the development of left ventricle hypertrophy in spontaneously hypertensive rats^14^. Moreover, inhibition of EGFR by specific tyrosine kinase inhibitor AG1478 significantly decreases the angiotensin II-mediated synthesis of Transforming Growth Factor (TGF)-β and fibronectin by cardiac fibroblasts^15^. Recently, our group found that EGFR inhibition significantly attenuated streptozotocin-induced diabetic heart injuries^16^. These studies suggest that EGFR antagonism may be an effective drug target for cardiac hypertrophy and remodeling. However, it is unknown if EGFR play a role in the pathogenesis of obesity-related cardiac injury.

The aim of this study was to determine the role of EGFR in hyperlipidemia-induced cardiac damage. EGFR was pharmacologically inhibited by two previously reported small-molecule inhibitors, AG1478 and 542 (Fig. 1A)^17^, or genetically silenced by siRNA in animal and cellular models. Inhibition of EGFR phosphorylation by AG1478 or 542 reduced myocardial inflammation, fibrosis, hypertrophy, apoptosis, and dysfunction in high fat diet-fed mice. Our results contribute to understand the detrimental role and mechanism of EGFR activation in obesity-related cardiac disorders.

## Results

### Small-molecule EGFR Inhibitors Attenuated HFD-induced Cardiac Injury and Dysfunction in apolipoprotein E knockout (ApoE^−/−^) Mice

**Figure 1:**
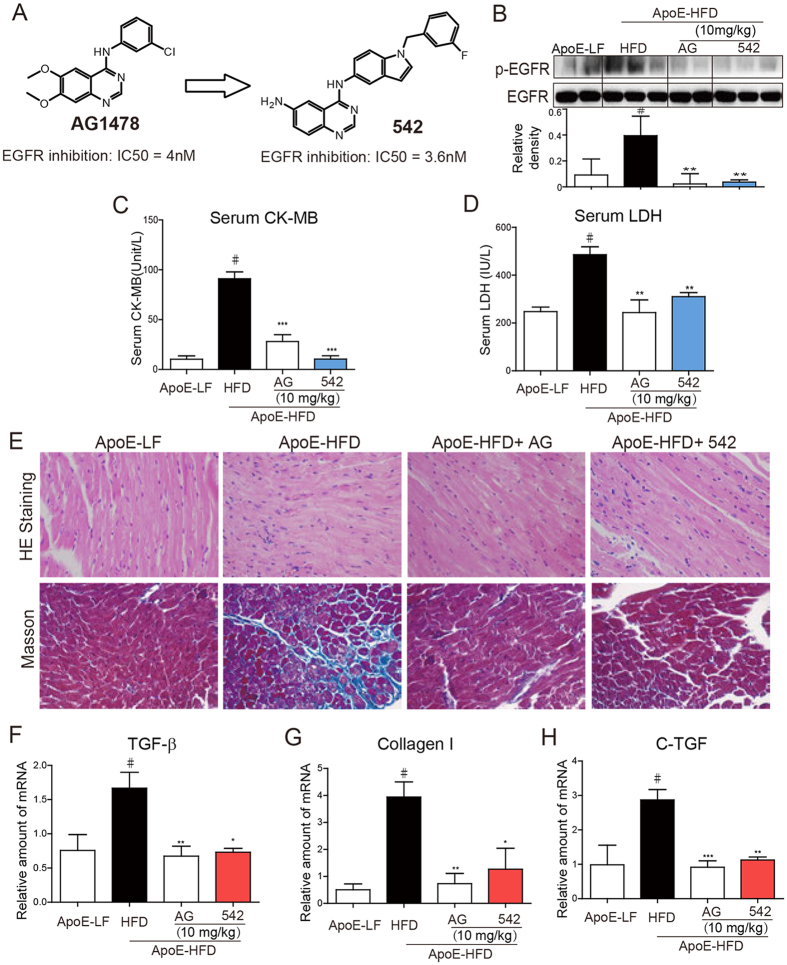
Small molecule EGFR inhibitors attenuate HFD-induced EGFR phosphorylation and myocardial fibrosis in ApoE^−/−^ mouse hearts. (**A**) Chemical structures and EGFR-inhibitory IC_50_ values of AG1478 and 542. ApoE^−/−^ mice were fed with HFD for 8 weeks, and then administrated with AG1478 (AG, 10 mg/kg/day) or 542 (10 mg/kg/day) for 8 Weeks in oral (n = 7 per group). (**B**) Total proteins extracted from heart tissues were subjected to Western blot analysis for EGFR phosphorylation level. Representative Western blots from randomly 2/3 mice per group were shown and the column figure showed the statistical results from the Western blot analysis of 7 mice per group. The gels were run under the same experimental conditions. Shown are cropped gels/blots (The gels/blots with indicated cropping lines are shown in Supplementary Fig. S4). Serum CK-MB (**C**) and LDH (**D**) levels were detected using a convenient kit (n = 7 per group). Representative pictures of H&E staining and Masson staining of heart tissue are shown. (**E**) Heart tissues from mice were individually processed for RNA extraction and real-time quantitative PCR. The mRNA levels of TGF-β (**F**), collagen I (**G**) and CTGF (**H**) were determined (n = 7 per group). ^#^*p* < 0.05 vs. ApoE-LF group; *p < 0.05, **p < 0.01, ***p < 0.001 vs. APOE-HFD alone. All images were obtained by microscope with original magnification ×400.

ApoE^−/−^ mice were first fed with HFD for 8 weeks (ApoE-HFD), and then administrated with AG1478 (AG, 10 mg/kg/day) or 542 (10 mg/kg/day) for another 8 weeks by oral gavage. AG or 542 treatment blocked HFD induced cardiac EGFR phosphorylation *in vivo* (Fig. 1B), without affecting the plasma level of low density lipoprotein (LDL) and total triglyceride (TG) (Sup Fig. S1A–C). Serum levels of creatinine kinase MB isoenzyme (CK-MB), Lactate Dehydrogenase (LDH) were also decreased with the administration of AG or 542, indicating less cardiac injury (Fig. 1C,D). Mouse heart weight to tibial length ratio was increased under HFD (Table 1), and histological analysis by H&E and Masson staining of cardiac sections showed disordered cardiac muscle fibers and increased myocardial fibrosis in ApoE-HFD heart, suggesting cardiac hypertrophy and remodeling. Treatment of AG or 542 decreased heart weight to tibial length ratio (Table 1), and reversed cardiac fibrosis induced by diet composition (Fig. 1E). This histological change was further confirmed by molecular marker analysis. The cardiac gene expression levels of fibrotic factors, including TGF-β, Collage I and connective tissue growth factor (CTGF), were all decreased upon AG or 542 treatment compared to ApoE-HFD alone. Furthermore, functional analysis by echocardiography demonstrated that AG or 542 prevented left ventricle dilation, and restored cardiac contractile function of ApoE-HFD hearts (Table 1). Taken together, small-molecule EGFR inhibitors AG and 542 attenuate HFD-induced cardiac injury and dysfunction in ApoE^−/−^ hearts.

### EGFR Inhibitors Suppressed HFD-induced Inflammation in ApoE^−/−^ Mouse Hearts

**Figure 2:**
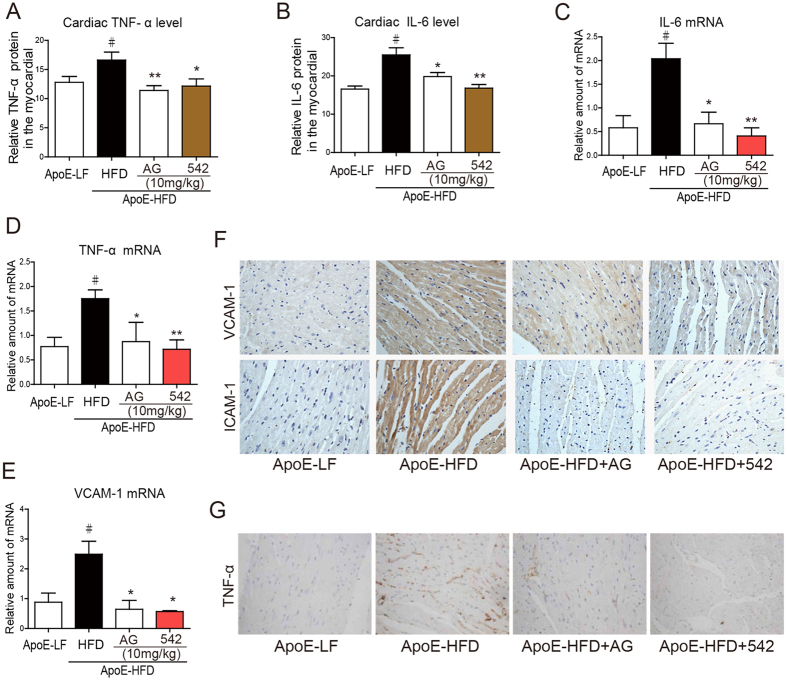
542 or AG1478 suppress HFD-induced inflammation in ApoE^−/−^ mouse hearts. (**A,B**) TNF-α and IL-6 protein levels in the heart tissues were detected using ELISA assay, as described in the methods (n = 7 per group for determination). (**C–E**) AG1478 or 542 administration reduced IL-6, TNF-α and VCAM-1 gene expression in APOE-HFD hearts. Heart tissues from each group were individually processed for RNA extraction and real-time quantitative PCR. The mRNA levels of IL-6, TNF-α and VCAM-1 were normalized to β-actin (n = 7). (**F**–**H**) Representative immunohistochemical images for VCAM-1 and ICAM-1 (**F**), and TNF-α (**G**) expression in heart tissue are shown. All images were obtained by microscope with original magnification (×400). ^#^*p* < 0.05 vs. ApoE-LF group; *p < 0.05, **p < 0.01 vs. ApoE-HFD alone.

Hyperlipidemia also causes substantial inflammatory response in the heart, which mediates the pathogenesis of cardiac injury and remodeling. We examined the expression level of pro-inflammatory cytokines. HFD increased the cardiac expression of TNF-α and IL-6 in ApoE^−/−^ hearts, as validated by both mRNA and protein levels (Fig. 2A–D). Administration of AG or 542 at 10 mg/kg for 8 weeks significantly reduced TNF-α and IL-6 protein expression in ApoE-HFD hearts (Fig. 2A–D). The mRNA level of vascular cell adhesion molecule 1 (VCAM-1), of which its upregulation occurs in response to HFD, was also decreased by AG or 542 treatment (Fig. 2E). Immunohistochemistry staining analysis also confirmed that treatment with AG or 542 remarkably reduced VCAM-1 and intercellular cell adhesion molecule 1 (ICAM-1) over-expression in ApoE-HFD hearts (Fig. 2F). This inhibitory effect of AG or 542 on cardiac tissue inflammation was further revealed by immunohistochemistry staining of TNF-α (Fig. 2G). The data together suggested that AG and 542 suppressed HFD-induced inflammation in the ApoE-HFD hearts.

### EGFR Inhibitors Reversed HFD-induced Cardiac Inflammation and injury in C57BL/6 Mice

**Figure 3:**
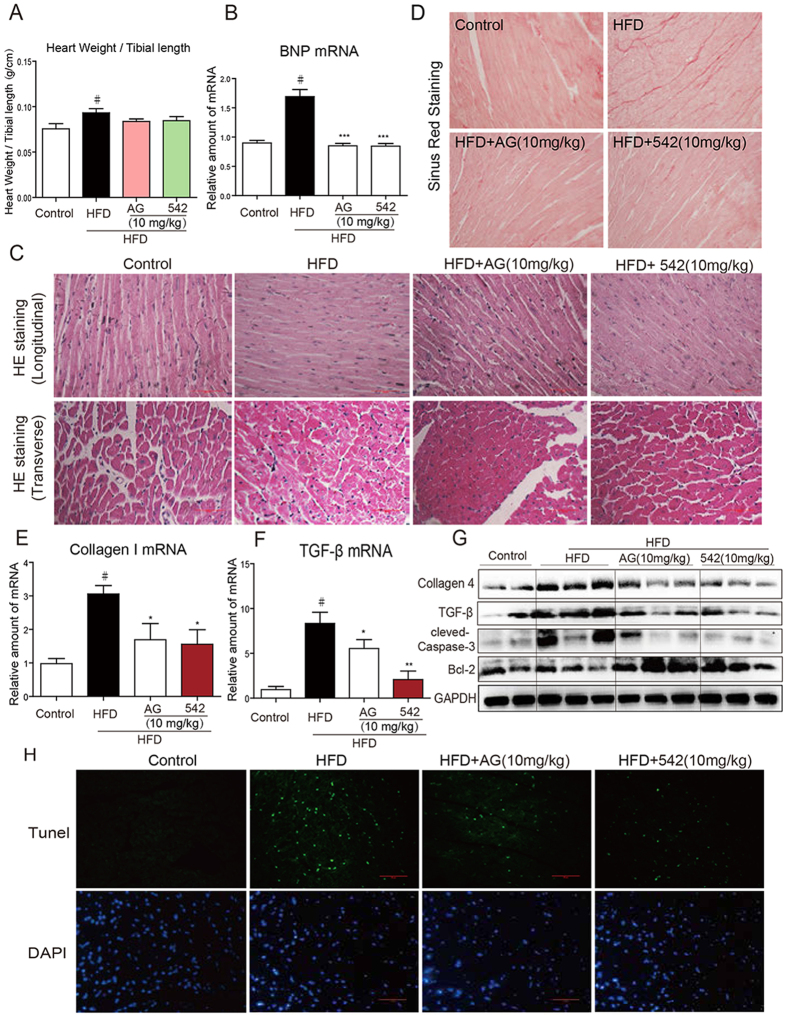
EGFR inhibitors reverse HFD-induced hypertrophic remodeling, fibrosis and apoptosis in C57BL/6 mouse heart. C57BL/6 WT mice were fed with HFD for 8 weeks, and treated with AG1478 (AG, 10 mg/kg/day) or 542 (10 mg/kg/day) for 8 weeks by oral gavage. (**A**) Heart Weight/Tibial length ratio (n = 7 per group). (**B**) qPCR analysis of BNP mRNA levels in the hearts (n = 7 per group). (**C**) Myocardial histological analysis was performed using H&E staining and representative images were shown (×400). (**D**) Myocardial fibrosis analysis was detected using Sirius Red staining and representative images were shown. qPCR analysis of TGF-β (**E**) and collagen1 (**F**) mRNA levels (n = 7 per group,). (**G**) The protein levels of collagen 4, TGF-β, cleaved-caspase-3, Bcl-2 were measured by western blot (n = 2 in control group; n = 3 in other groups). The gels were run under the same experimental conditions. Shown are cropped gels/blots (The gels/blots with indicated cropping lines are shown in Supplementary Fig. S4). (**H**) Representative images of TUNEL/DAPI staining of myocardium were shown. All images were obtained by microscope with original magnification (×400). ^#^*p* < 0.05 vs. control group; *p < 0.05, **p < 0.01, ***p < 0.001 vs. HFD alone.

**Figure 4:**
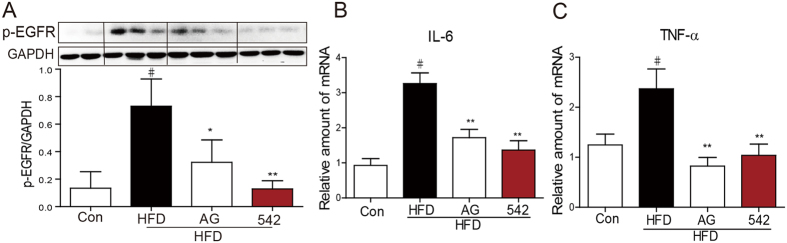
EGFR inhibitors decrease HFD-induced inflammation in C57BL/6 mouse heart. (**A**) The myocardial protein phosphorylation level of EGFR was measured by Western blot method and normalized to GAPDH (Shown are randomly selected mouse samples from four groups; n = 2 in control group, n = 3 in other groups. For the statistic column figure, heart tissues from all 7 mice in each group were used for western blot assay and density statistics; *p < 0.05 and **p < 0.01 vs. HFD). The gels were run under the same experimental conditions. Shown are cropped gels/blots (The gels/blots with indicated cropping lines are shown in Supplementary Fig. S4). (**B,C**) The cardiac mRNA levels of IL-6 (**B**), TNF-α (**C**) were assessed by qPCR and normalized to β-actin (n = 7; ^#^*p* < 0.05 vs. control group; *p < 0.05, **p < 0.01 vs. HFD alone).

To further validate the effect of EGFR inhibitors against hyperlipidemia-induced cardiomyopathy, we performed similar experiment in C57BL/6 WT mice. C57BL/6 mice were first fed with HFD for 8 weeks, and then administrated with AG1478 (AG, 10 mg/kg/day) or 542 (10 mg/kg/day) for 8 weeks by oral gavage. Serum lipid contents including LDL and TG were elevated in response to HFD (Sup Fig. S2A,B). Body weight and heart weight to tibial length ratio were increased by HFD treatment alone, but not in HFD plus EGFR inhibitor treatment (Fig. 3A, Sup Fig. S2C). Administration of AG or 542 markedly decreased the cardiac expression of B-type natriuretic peptide (BNP), a myocardial hypertrophy marker, when exposed to HFD (Fig. 3B), indicating that AG or 542 protects heart from hypertrophy and remodeling. H&E staining of both longitudinal and transverse cardiac sections showed myocardial fiber disorder and hypertrophy in HFD-fed mice, and AG or 542 was able to reverse this phenotype (Fig. 3C). Sirius Red staining revealed an obvious collagen accumulation in the HFD hearts, and EGFR inhibitors notably attenuated collagen deposition (Fig. 3D). Up-regulation of cardiac collagen 4 and TGF-β protein expression in response to HFD was also blocked by AG or 542 treatment (Fig. 3E–G), indicating that EGFR inhibition prevents cardiac fibrosis and remodeling in HFD mice. Meanwhile, AG or 542 reduced cleaved caspase-3 level in the heart while promoted anti-apoptotic protein Bcl-2 expression (Fig. 3G), which led to less cardiac apoptosis assessed by TUNEL and DAPI staining (Fig. 3H). On the other hand, HFD induced EGFR phosphorylation by about 4 fold. Administration of AG1478 and 542 remarkably decreased p-EGFR level in HFD mouse hearts (Fig. 4A). Further assessment of cardiac inflammation showed a significant reduction of TNF-α, IL-6 mRNA expression by AG or 542 treatment (Fig. 4B,C). Taken together, EGFR inhibition reversed HFD-induced myocardial hypertrophy, fibrosis, apoptosis and inflammation in mice.

### EGFR Inhibition Decreased Palmitic acid (PA)-induced Inflammation in H9C2 Cells

**Figure 5:**
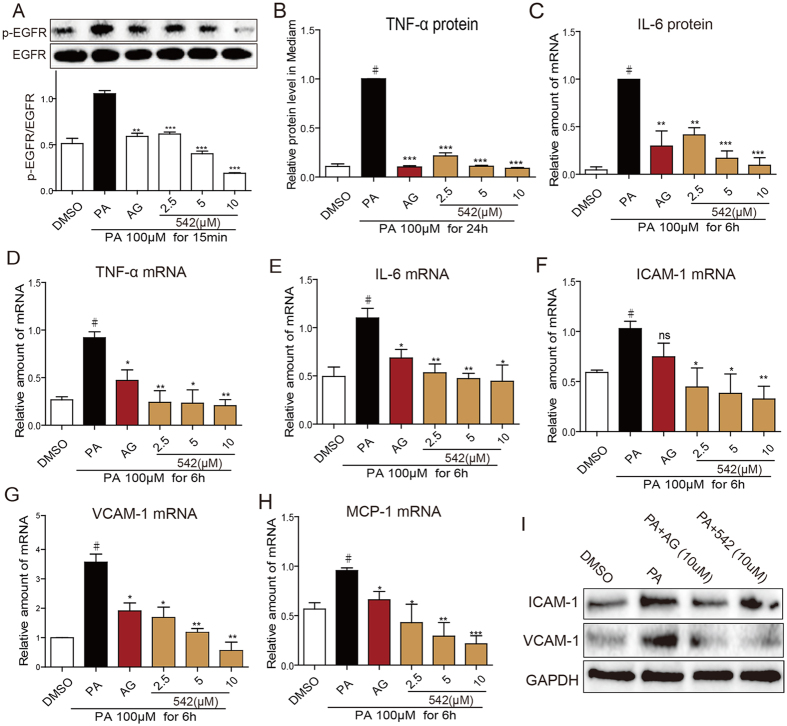
EGFR inhibitors attenuate PA-induced inflammation in H9C2 Cells. (**A**) H9C2 cells were pretreated with AG1478 (AG, 10 μM) or 542 (2.5, 5, 10 μM) for 2 h, and then incubated with PA (Palmitate, 100 μM) for 15 min. The p-EGFR level was detected by western blotting. The western blots were cropped from the same gel that was run under the same experimental conditions. The column figures show the normalized optical density for the data from three independent experiments. (**B,C**) H9C2 cells were pretreated with AG1478 or 542 for 2 h, and then incubated with PA (100 μM) for 24 h. TNF-ɑ and IL-6 concentrations in the medium were detected via ELISA, respectively; 4 separate determinations. (D–H) H9C2 cells were pretreated with AG1478 or 542 for 2 h, and then incubated with PA (100 μM) for 6 h. The mRNA levels of TNF-ɑ (**D**), IL-6 (**E**), ICAM-1 (**F**), VCAM-1 (**G**), and MCP-1 (**H**) were detected by q-PCR and normalized by β-actin. Bars represent the mean ± SD of four independent experiments run in triplicate. (^#^*p* < 0.05 vs. DMSO group; *p < 0.05, **p < 0.01, ***p < 0.001 vs. PA treatment alone). (**I**) H9C2 cells were pretreated with AG1478 or 542 at 10 μM for 2 h, and then incubated with PA (100 μM) for 24 h. The levels of ICAM-1 and VCAM-1 in cell lysate were detected via western blot analysis. Shown are representative blots from 3 separate determinations. The gels were run under the same experimental conditions. Shown are cropped gels/blots (The gels/blots of 5A and 5I with indicated cropping lines are shown in Supplementary Fig. S4).

EGFR is ubiquitously expressed in multiple cell types within cardiovascular system, including cardiomyocyte, smooth muscle cell and vascular endothelial cell^12^,^18^. To evaluate whether the cardioprotection of AG or 542 observed in the *in vivo* model is a direct effect on cardiomyocytes, we pretreated H9C2 cells with AG (10 μM) or 542 (2.5, 5, 10 μM) for 2 h, and then incubated with PA (100 μM) for various time points. PA stimulation for 15 min increased EGFR tyrosine phosphorylation by about two fold in H9C2 cells (Fig. 5A) and EGFR inhibitor 542 reduces EGFR phosporylation in a dose-dependent manner (Fig. 5A). Upon sustained PA treatment for 24 h, the TNF-ɑ and IL-6 protein secretion were stimulated, which were dose-dependently reduced by 542 pretreatment (Fig. 5B,C). Also, 542 suppressed the PA-induced inflammatory gene expression, such as TNF-ɑ, IL-6, ICAM-1, VCAM-1, and monocyte chemoatt ractant protein (MCP)-1 in a dose-dependent manner (Fig. 5D–H). The inhibition of PA-increased expression of adhesion molecules ICAM-1 and VCAM-1 by 542 or AG pretreatment were further observed by western blot assay (Fig. 5I). These data suggested that EGFR inhibitors directly act on cardiomycoyte to exert the anti-inflammatory effect.

### EGFR Inhibitors Prevented PA-induced Injury in H9C2 Cells

**Figure 6:**
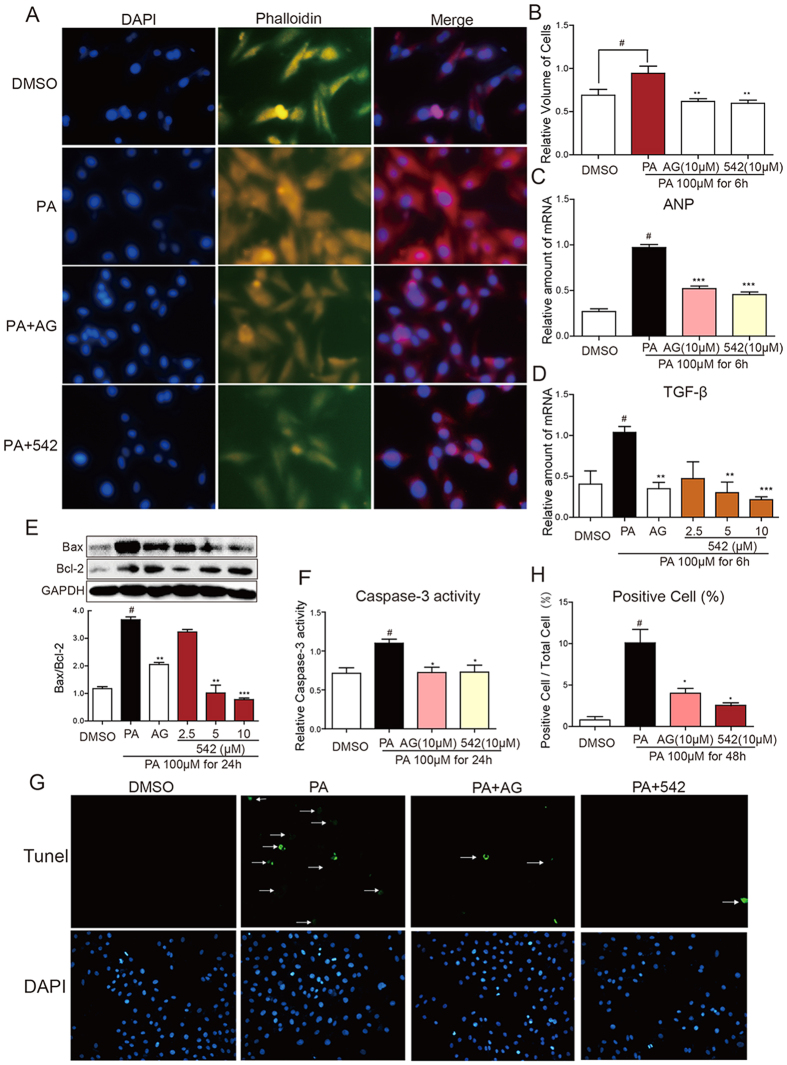
EGFR inhibitors reverse PA-induced hypertrophy, fibrosis and apoptosis in H9C2 cells. H9C2 cells were pretreated with AG1478 (AG, 10 μM) or 542 (10 μM) for 2 h, and then exposured to PA (100 μM) for indicated time. (**A,B**) Phalloidin/DAPI staining and cell volume measurement of H9C2 cells with PA (100 μM) treatment for 6 h. The column figure (**B**) show the quantitative cell sizes from three independent experiments. (**C,D**) Exposing to PA (100 μM) for 6 h, the mRNA levels of TGF-β and ANP were detected by real-time qPCR and normalized by β-actin. Bars represent the mean ± SD of four independent experiments run in triplicate. (**E**) Bcl-2 and Bax protein levels, and (**F**) the caspase-3 activity were determined in cells with PA (100 μM) treatment for 24 h. The gels were run under the same experimental conditions. Shown are cropped gels/blots (The gels/blots with indicated cropping lines are shown in Supplementary Fig. S4). The columns show the normalized optical density for data from three independent experiments. (**G,H**) Representative pictures and quantitative analysis of three independent TUNEL/DAPI staining after exposure to PA (100 μM) for 48 h. Values are reported as means ± SEM (n = 4, ^#^P < 0.05, ^##^P<0.01, vs. DMSO group; *P < 0.05, **P<0.01, ***P<0.001, vs. PA group).

To evaluate the effect of EGFR inhibitors on cardiomyocyte hypertrophy *in vitro*, H9C2 cells were pretreated with AG1478 (AG, 10 μM) or 542 (10 μM) for 2 h, and then exposed to PA (100 μM) for 6 h. Rhodamine-labled Phalloidin was used to assess the cell morphology and cell volume. Incubation of PA significantly increased H9C2 cell volume, while AG or 542 can reverse the cell morphology change (Fig. 6A,B). Meanwhile, the mRNA level of hypertrophic marker gene atrial natriuretic peptide (ANP) and profibrotic gene TGF-β in H9C2 cells were markedly decreased by AG or 542 pretreatment (Fig. 6C,D), suggesting these small molecule inhibitors prevent PA-induced cardiomyocyte hypertrophy and fibrosis.

We further tested the protective effect of AG or 542 in H9C2 cells. Incubation of PA for 48 h significantly triggers H9C2 cell apoptosis, as evidenced by the increased TUNEL positive cells, elevated Bax protein expression and caspase-3 activity (Fig. 6E,H). Pretreatment of AG or 542 decreased Bax/Bcl-2 protein ratio in a dose dependent manner, and also reduced intracellular caspase-3 activity (Fig. 6E,F). As a result, the TUNEL positive cells were also decreased by AG or 542 treatment (Fig. 6G,H). Taken together, these results suggest that inhibition of EGFR phosphorylation by small molecules reversed PA-induced cardiomyocyte injuries.

### Genetic knockdown of EGFR Inhibited PA-induced Inflammatory Injury in H9C2 Cells

**Figure 7:**
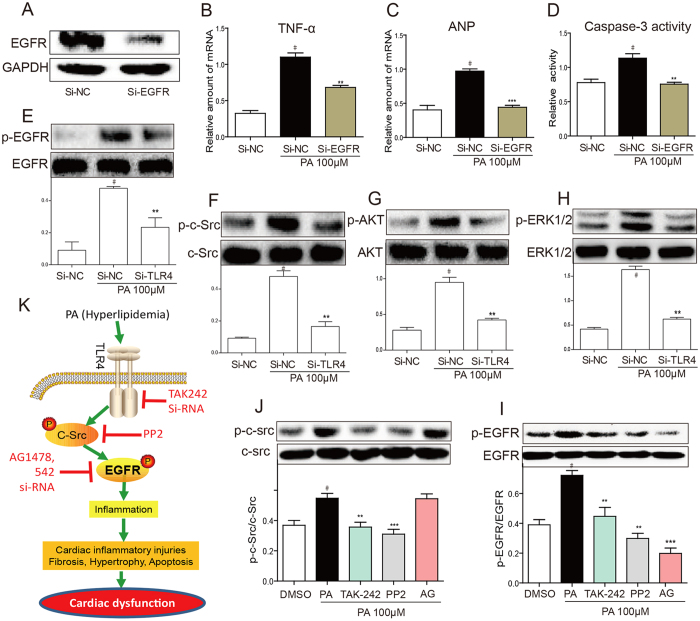
PA induces EGFR activation through TLR4/c-Src signaling cascade in H9C2 cells. (**A–D**) EGFR siRNA attenuated PA-induced cell injury. H9C2 cells were pretreated with EGFR siRNA for 6 h, and the down-regulation of EGFR protein expression was determined by western blotting (**A**). A scrambled sequence for the EGFR siRNA was used as the negative control (si-NC). Similar results were observed in three independent experiments. After incubating for 24 h, EGFR-silencing H9C2 cells were stimulated with PA (100 μM) for 6 h. TNF-ɑ (**B**) and ANP (**C**) mRNA levels were measured by qPCR and normalized by β-actin. Bars represent the mean ± SD of four independent experiments run in triplicate. The intracellular caspase-3 activity was evaluated upon PA (100 μM) treatment for 24 h (**D**). (**E–H**) TLR4 siRNA inhibited PA-induced EGFR pathway activation. H9C2 cells were pretreated with siTLR4 for 6 h to knock down TLR4. The phosphorylation levels of EGFR (**E**), c-Src (**F**), AKT (**G**), and ERK1/2 (**H**) were determined by western blot method after PA (100 μM) treatment for 15 min. The column figure shows the normalized optical density from four independent experiments. (**I,J**) H9C2 cells were pretreated with TAK-242 (TLR4 inhibitor), or PP2 (c-Src inhibitor), or AG1478 for 1 h, followed by PA (100 μM) treatment for 15 min. Phosphorylation of EGFR (**I**) and c-Src (**J**) were determined by western blot analysis. The gels were run under the same experimental conditions. Shown are cropped gels/blots (The gels/blots of 7**A**,**E**–**J** with indicated cropping lines are shown in Supplementary Fig. S4). The column figure shows the normalized optical density from four independent experiments. Values are reported as means ± SEM (n = 4, ^#^P < 0.05, vs. si-NC or DMSO group; **P < 0.01, ***P < 0.001, vs. PA treatment group). (**K**) A schematic illustration of the molecular mechanism underlying the protection of EGFR inhibitors against hyperlipidemia-induced lipotoxicity cardiomyopathy.

To avoid the non-specificity of small-molecule inhibitors and validate the role of EGFR, we transfected the H9C2 cells with specific small-interfering RNA to down-regulate EGFR expression (si-EGFR). As shown in Fig. 7A, transfection of si-EGFR under 100 μM PA treatment significantly decreases EGFR protein expression in H9C2 cells, which further led to a decreased gene expression level of TNF-ɑ and ANP, and reduced caspase-3 activity in PA-stimulated H9c2 cells (Fig. 7B–D). These results, together with our observation of the intracellular effect of 542/AG1478, confirmed that EGFR plays an important role in mediating hyperlipidemia-induced cardiac injury. To mimic the clinical setting, we further evaluated the protective effects of EGFR inhibition against the deleterious actions of PA in H9c2 cells, which were already exposed to PA (as a treatment). The results in the supplementary Fig. S3 showed that post-treatment with EGFR inhibitors AG or 542 also decreased PA-increased TNF-α and ANP levels.

### PA Induces EGFR Activation Through TLR4/c-Src Signaling Cascade in H9C2 Cells

It remains unclear how fatty acid activates EGFR. Previous study demonstrated that fatty acid directly activates Toll-like receptor 4 (TLR4)^19^,^20^, which further induces c-Src kinase activation^21^,^22^. Thus we speculated that lipids trigger EGFR signaling pathway through TLR4/c-Src signaling cascade. H9C2 cells were transfected with TLR4 siRNA (si-TLR4) to knock down TLR4 protein expression. The si-TLR4 blocked the phosphorylation of c-Src and EGFR induced by PA treatment (Fig. 7E,F), therefore, the downstream signaling Akt and ERK phosphorylation has been reduced (Fig. 7G,H). We also pretreated H9C2 cells with TLR4 inhibitor (TAK-242) and c-Src inhibitor (PP2) before stimulation with PA. Pretreatment of both TAK-242 and PP2 decreases phosphorylation level of c-Src and EGFR, while AG pretreatment can only block EGFR phosphorylation but has no effect on c-Src (Fig. 7I,J). These results together suggested that TLR4/c-Src signaling mediated PA-triggered EGFR activation.

## Discussion

Obesity has become an epidemic now, and represents one of the most prevalent disorders. Cardiovascular risks are the major cause of mortality and morbidity in patients with obesity. Despite there were many proposed mechanisms, the scientific community has yet to reach a definitive consensus about the underlying mechanisms, involved in the cardiac functional and morphological changes associated with obesity. Here in this study, we have demonstrated that satuarated fatty acid (SFA) triggers the phosphorylation and activation of EGFR in both H9C2 cells and *in vivo* cardiac tissues, which further led to cardiac inflammation and fibrosis. Application of EGFR inhibitors, 542 and AG1478, on either PA-challenged cells or HFD-treated animals showed a great reduction of cardiac inflammatory injuries. AG1478 and 542 also attenuated cardiac dysfunction in HFD-fed ApoE^−/−^ mice, suggesting that EGFR antagonism may be a therapeutic strategy for obesity-induced heart injury.

Recently, it has been reported that EGFR plays an important role in cardiac remodeling in response to extracellular stimuli. In a mice model of myocardial ischemia, the interaction between heparin-binding EGF (HB-EGF) and EGFR transactivation is closely related to the proliferation of cardiac fibroblasts and cardiac remodeling^23^. Galan *et al.* reported that enhanced EGFR phosphorylation and its downstream ER stress is involved in cardiac fibrosis and microvascular endothelial dysfunction in type I diabetes mellitus^24^. A direct role for EGFR in remodeling is supported by the observation that EGFR mediates angiotensin II-induced expression of TGFβ and fibronectin in cultured rat cardiac fibroblasts^15^. Overexpression of dominant negative EGFR or EGFR tyrosine kinase inhibitor AG1478 dramatically reduced TGF-β and fibronectin expression in cultured cells^15^, suggesting a pro-fibrotic role of EGFR signaling. Here in our study, we observed a similar effect in both ApoE^−/−^ and C57BL/6 mice of HFD treatment. Administration of EGFR inhibitors significantly inhibited EGFR phosphorylation, decreased the expression of TGF-β, collagen I and CTGF in the myocardium of animals subjected to HFD, which led to less cardiac fibrosis and dysfunction.

EGFR also contributes to hyperlipidemia-induced cardiac inflammation. Lipid overload is often associated with increased production and release of pro-inflammatory cytokines, such as TNF-α, IL-6 and MCP-1. The inflammatory cytokines activate a series of intracellular signaling pathways including nuclear factor (NF)-κB and JNK, which up-regulate the transcription of more cytokines and exaggerate the inflammatory response. These events further trigger macrophage activation, migration and tissue filtration, leading to tissue injury^25^,^26^. Inhibition of EGFR may be a therapeutic strategy for reducing inflammation injury. It was reported that EGFR inhibitor Gefitinib reduces airway inflammation^27^. As observed in our study, EGFR plays a crucial role in PA-induced inflammation and apoptosis. Treatment of EGFR inhibitors AG1478 and 542 in H9C2 cells significantly decreased TNFα and IL-6 expression. In HFD-induced cardiac injury mouse models, EGFR inhibitors also effectively reduced tissue inflammation in the heart, and reversed the changes in cardiovascular structure and function. The anti-inflammatory effects of EGFR inhibition were validated using siRNA-silencing EGFR knockdown in H9c2 cells. These *in vitro* and *in vivo* data demonstrate the strong anti-inflammatory effect of EGFR antagonism, which may contribute mainly to its cardioprotective effects.

The activation of EGFR is not directly mediated by saturated fatty acid (SFA). Recent studies show that only unsaturated fatty acids triggered tyrosine phosphorylation of EGFR in endothelial cells, whereas saturated FAs were inactive^28^. Similarly, another study revealed that unsaturated fatty acid, but not saturated fatty acid, was able to perturb cellular EGFR transmembrane signaling in EGFR T17 cloned cell line, indicating that SFA cannot directly bind to EGFR^29^. However, several laboratories have independently reported that the intracellular effects of SFAs are mediated at least in part, through activation of TLR4^30^, suggesting that TLR4 may be a mediator of SFA-induced EGFR activation. In our cultured H9C2 cells, when silencing TLR4 gene expression by siRNA approach, EGFR phosphorylation was dramatically decreased during SFA treatment, indicating that TLR4 is the upstream regulator of EGFR (Fig. 7E–H). The mechanism of how TLR4 regulates EGFR activation falls into two main categories: 1) TLR4 colocalized with EGFR to form a complex on the cell membrane^31^, and it transactivates EGFR by Src kinase^21^,^22^; 2) TLR4 stimulate the expression of EGFR endogenous ligands, which in turn activates EGFR^32^. As palmate acid acutely (within 15 min) triggers EGFR phosphorylation in H9C2 cells, it is less likely that this was through the EGFR ligand expression. Consistently, our date showed that PA increased c-Src phosphorylation, while TLR4 inhibitor TAK-242 inhibited TLR4 dimerization and reduced c-Src Phosphorylation. Moreover, through inhibition of c-Src kinase activity, PP2 inhibits EGFR activation under PA stimulation. Our data suggest that EGFR mediates PA-induced inflammation, involving TLR4 and c-Src activation. Recently, G. Stark’s group demonstrated the mechanism of involvement of EGFR in lipopolysaccharide (LPS)-TLR4 pro-inflammatory signaling pathway^33^. They found that a cross talk between TLR4 and EGFR triggers NF-κB activation. LYN proto-oncogene (LYN), a member of Src family, is required for TLR4-EGFR signaling in both directions to NF-κB. These data together indicate that the TLR4-c-Src-EGFR signaling mediates the pro-inflammatory induction of PA/obesity, providing a pathway for SFA-induced EGFR activation and inflammation (Fig. 7K).

In summary, this study has confirmed the detrimental effect of EGFR activation in the pathogenesis of obesity-induced cardiac inflammatory injuries in experimental mice, and has demonstrated the TLR4/c-Src-mediated mechanisms for PA-induced EGFR signaling pathway activation in H9c2 cells. Blocking EGFR phosphorylation by small molecules demonstrates the beneficial effect on cardioprotection against remodeling and dysfunction. Our data suggest that EGFR may be a therapeutic target for obesity-related cardiovascular diseases, and that the novel EGFR inhibitor 542 shows promises to the treatment on obesity-induced cardiac complications.

## Methods

### Reagents

Palmitate (PA), PP2, TAK-242 and AG1478 were purchased from Sigma-Aldrich (St. Louis, MO, USA). Novel EGFR inhibitor 542 was synthesized and characterized by our group. 542 and AG1478 were dissolved in DMSO for in *vitro* experiments and in sodium carboxyl methyl cellulose (CMC-Na) (1%) for *in vivo* experiments. Actived-Caspase-3 kit, Enhanced chemiluminescence reagent, and One Step TUNEL Apoptosis Assay Kit were obtained from Beyotime (Nantong, China).

### Cell Culture and treatment

Embryonic rat heart-derived cell line H9C2 was obtained from the Shanghai Institute of Biochemistry and Cell Biology (Shanghai, China). H9C2 was cultured in DMEM/F12 medium (Gibco, Eggenstein, Germany) containing 2.25 g/L glucose medium supplemented with 10% FBS, 100 U/ml of penicillin, and 100 mg/ml of streptomycin in a humidified atmosphere of 5% CO_2_ at 37 °C. For *in vitro* studies, stock solutions of 5mM PA/10% fatty acid-free BSA were prepared and stored at −20 °C. Stock solutions were heated for 15 min at 55 °C and then cooled to room temperature prior to use. The fatty acid-BSA complex was added to the serum-containing cell culture medium to achieve a fatty acid concentration of 100 μM.

### Animals

This investigation conforms to the guidelines of NIH on the protection of animals used for scientific purposes. Male C57BL/6 mice weighing 18–20 g aged 8 weeks were purchased from SLAC Laboratory Animal Center (Shanghai, China). Male ApoE^−/−^ mice weighing 18–20 g aged 8 weeks were purchased from HFK Bioscience Co. LTD (Beijing, China). Animals were housed at a constant room temperature with a 12:12 h light-dark cycle and fed with a standard rodent diet and water. The animals were acclimatized to the laboratory at least for 3 days before used. Animal protocols were approved by the Wenzhou Medical University Animal Policy and Welfare Committee. All animal care and experiments were performed in accordance with the approved protocols and the ‘The Detailed Rules and Regulations of Medical Animal Experiments Administration and Implementation’ (Document No. 1998–55, Ministry of Public Health, China).

### High Fat Diet-fed Animal Experiments

After an acclimatization period of one week, 28 C57BL/6 or ApoE^−/−^ mice were randomly divided into four weight-matched groups. 7 mice were fed with standard animal low-fat diet containing 10 kcal.% fat, 20 kcal.% protein and 70 kcal.% carbohydrate (MediScience Diets Co. LTD, Yangzhou, China, Cat. #MD12031) served as a normal control group (Control or ApoE-LF), while the remaining 21 mice were fed with high-fat diet containing 60 kcal.% fat, 20 kcal.% protein and 20 kcal.% carbohydrate (HFD, MediScience Diets Co. LTD, Yangzhou, China, Cat. #MD12033) for 16 weeks. Since 9^th^ week, AG1478 or 542 were given daily by oral gavage at a dose of 10 mg/kg/day for the next 8 weeks. Mice in the Control and HFD groups were gavaged with vehicle (1% CMC-Na solution) only. At the day before the sacrifice of ApoE^−/−^ mice, doppler analysis was performed to determine the pathologic cardiac hypertrophy. Mice was anesthetized with isoflurane, echocardiography was performed by SONOS 5500 ultrasound (Philips Electronics, Amsterdam, Netherland) with a 15-MHz linear array ultrasound transducer. At the end of experimental period, all the animals were sacrificed by cervical decapitation. The body weight was recorded. Blood samples were collected and centrifuged at 4 °C at 3000 rpm for 10 min for collecting the serum. The heart was excised aseptically, blotted dry and the weight was recorded followed by immediate freezing in liquid nitrogen and then stored at −80 °C before further analyses.

### Western Blot Analysis

Cell lysate was prepared from cultured cells or homogenized myocardial tissue samples. 50–90 mg of lysates were separated by 10% SDS-PAGE and electrotransferred to a nitrocellulose membrane. Each membrane was pre-incubated for 1 h at room temperature in Tris-buffered saline, pH 7.6, containing 0.05% Tween 20 and 5% non-fat milk. Each nitrocellulose membrane was incubated with specific antibodies. Antibodies against TGF-β, Collagen 4, Bax, Bcl2, Caspase-3 and p-c-Src, c-Src, GAPDH were purchased from Santa Cruz Technology (Santa Cruz, CA). Antibodies against p-EGFR Tyr835, EGFR, p-AKT, AKT, p-ERK1/2, and ERK1/2 were from Cell Signaling Technology (Danvers, MA). Immunoreactive bands were then detected by incubating with secondary antibody conjugated with horseradish peroxidase, and visualized using enhanced chemiluminescence reagents (Bio-Rad, Hercules, CA). The amounts of the proteins were analyzed using Image J, and normalized to their respective control.

### Real-time Quantitative PCR

Total RNA was isolated from cells or tissues (50–100 mg) using TRIZOL (Invitrogen, Carlsbad, CA). Reverse transcription PCR (RT-qPCR) were performed using M-MLV Platinum RT-qPCR Kit (Invitrogen, Carlsbad, CA). Real-time qPCR was carried out using the Eppendorf Real plex 4 instrument (Eppendorf, Hamburg, Germany). Primers for genes including TNF-α, IL-6, ICAM-1, MCP-1, Collagen1, TGF-β, ANP, BNP, and β-actin were synthesized in Invitrogen (Invitrogen, Shanghai, China). The primer sequences used were shown in Table S1. The relative amount of each gene was normalized to the amount of β-actin. And, MIQE guidelines were followed in the real-time qPCR experiments.

### siRNA-induced Gene Silencing

Silencing gene expression was achieved using the siRNA technique. EGFR siRNA was purchased from Gene Pharama (Shanghai, China). Transfection of H9C2 cells with siRNAs was carried out using LipofectAMINE™ 2000 (Invitrogen, Carlsbad, California), according to the manufacturer’s instruction. Transfected cells were then treated with Palmitate for the following experiments. Specific siEGFR Sequences were as follows: sense (5′-3′) 5-CCGUGCCUGAAUAUAUAAATT-3; antisense (5′-3′) 5-UUUAUAUAUUCAGGCACGGTT-3.

### Measurement of Cell Surface Area

The cell culture media was aborted and the cells were washed with PBS and fixed with 4% paraformaldehyde and permeabilised with 0.1% Triton-X100, and then the cells were stained with rhodamine phalloidin at a concentration of 50 μg/ml for 30 min at RT and then washed with PBS and detected by fluorescence microscope.

### Inflammatory Cytokines Secretion Detection Using ELISA

After treatment, cell culture medium or Homogenized myocardial tissue samples were collected and 100 ul was processed for ELISA using a human TNF-ɑ or IL-6 ELISA detection kit from eBioscience (San Diego, USA) according to the instruction of manufacturer recommended.

### Histopathology

Excised heart tissue specimens were fixed in 10% formalin processed in graded alcohol, xylene, and then embedded in paraffin. Paraffin blocks were sliced into sections of 5 μm in thickness. After rehydration, the sections were stained with Hematoxylin and Eosin (H&E). To evaluate the histopathological damage, each image of the sections was captured using a light microscope (400× amplification, Nikon, Japan). For each staining, totally 3 × 7 sections (7 mice) per group are observed and the representative images were shown in Figures, and all observations were done by a blinded reviewer.

### Massion and Sirius Red staining

Paraffin sections (5 um) were stained with 0.1% Sirius Red F3B and 1.3% saturated aqueous solution of picric acid to evaluate the type IV collagen deposition, or were stained with Masson’s trichrome (Sigma) for fibrosis. The stained sections were then viewed under the Niko fluorescence microscope (400× amplification; Nikon, Japan). For each staining, totally 3 × 7 sections (7 mice) per group are observed and the representative images were shown in Figures, and all observations were done by a blinded reviewer.

### Immunofluorescence assay for TNF-α

Paraffin sections (5 μm) were deparaffinized and rehydrated, and then subjected to antigen retrieval in 0.01 mol/L citrate buffer (pH 6.0) by microwaving. After blocking with 5% BSA, the sections were incubated with anti-TNF-α antibody (1:500) overnight at 4 °C, followed by the secondary antibody (1:200; Santa Cruz, CA). The nucleus was stained with Hematoxylin. The stained sections were then viewed under the Nikon fluorescence microscope (400× amplification; Nikon, Japan). For each staining, totally 3 × 7 sections (7 mice) per group are observed and the representative images were shown in Figures, and all observations were done by a blinded reviewer.

### TUNEL Staining

Tissue sections of 5 μm were used for the terminal deoxynucleotidyl transferase-mediated FITC-dUTP nick end labeling (TUNEL) apoptosis detection using one Step TUNEL Apoptosis Assay Kit acquired from Beyotime (Beijing, China) according to the manufacturer’s instructions. TUNEL positive cells were imaged under a fluorescence microscope (400× amplification; Nikon, Japan). For each staining, totally 3 × 7 sections (7 mice) per group are observed and the representative images were shown in Figures.

### Caspase-3 Activity

Cell lysate was prepared and detected using commercial kits (Byotime) according to the instruction of manufacturer recommended.

### Measurements of the Level of Serum Lipid

The components of serum lipid including the total triglyceride (TG), density lipoprotein (LDL), total cholesterol (TCH), CK-MB, and LDH were detected using commercial kits (Nanjing Jiancheng, Jiangsu, China).

### Statistical Analysis

Data were analyzed using Student’s t-test and multiple-comparison ANOVA in GraphPad Prism-5 statistic software (La Jolla, CA, USA). All of the values are presented as the mean ± standard error of the mean (SEM) from at least 4 separate experiments or determinations. A difference of p < 0.05 was considered to be statistically significant.

## Additional Information

**How to cite this article**: Li, W. *et al.* EGFR Inhibition Blocks Palmitic Acid-induced inflammation in cardiomyocytes and Prevents Hyperlipidemia-induced Cardiac Injury in Mice. *Sci. Rep.* **6**, 24580; doi: 10.1038/srep24580 (2016).

## Supplementary Material

Supplementary Information

## Figures and Tables

**Table 1:** 542/AG1478 treatment attenuated HFD-induced alterations of cardiac function in ApoE^−/−^ mice.

Parameter	ApoE-LF	ApoE-HFD	ApoE-HFD + AG	ApoE-HFD + 542
Heart weight (HW, mg)	149 ± 4	177 ± 4*	152 ± 7^#^	144 ± 10^#^
Tibial length (TL, mm)	20.8 ± 0.2	21.5 ± 0.1	21.6 ± 0.5	21.3 ± 0.2
HW/TL (mg/mm)	7.15 ± 0.17	8.92 ± 0.149*	7.03 ± 0.36^#^	6.95 ± 0.29^#^
Heart Rate(bmp)	517 ± 16	525 ± 28	554 ± 12	521 ± 16
LVEDD (mm)	2.17 ± 0.17	2.58 ± 0.09*	2.28 ± 0.09^#^	2.1 ± 0.08^#^
LVESD (mm)	1.16 ± 0.11	1.79 ± 0.06*	1.22 ± 0.07^#^	1.1 ± 0.06^#^
LVmass (mg)	41.5 ± 4.4	56.8 ± 6.3*	46.2 ± 5.2^#^	42 ± 3.9^#^
Fractional shortening (%)	47.0 ± 3.0	31.2 ± 1.5*	45.5 ± 2.3^#^	44.9 ± 1.4^#^
LVEF (%)	84.5 ± 2.9	61.6 ± 1.3*	82.5 ± 2.2^#^	82.9 ± 0.9^#^

Mean ± SEM, n = 7 per group; *p < 0.05 vs. ApoE-LF group; ^#^p < 0.05 vs. ApoE-HFD group. LV, left ventricle; LVEF, left ventricle ejection fraction; LVEDD, left ventricle end diastolic diameter; LVESD, left ventricle end systolic diameter.
