# Supplementary material for: EGFR Inhibition Blocks Palmitic Acid-induced inflammation in cardiomyocytes and Prevents Hyperlipidemia-induced Cardiac Injury in Mice
Source: Sci Rep. 2016 Apr 18;6:24580. doi: 10.1038/srep24580 (PMC5263857; doi:10.1038/srep24580)
Supplement: Supplementary Information [file srep24580-s1.doc]

**Supplementary information**

**EGFR Inhibition Blocks Palmitic Acid-induced inflammation in cardiomyocytes and Prevents Hyperlipidemia-induced Cardiac Injury in Mice**

Weixin Li^1,2,#^, Qilu Fang^1,#^, Peng Zhong^1^, Lingfeng Chen^1^, Lintao Wang^1^, Yali Zhang^1^, Jun Wang^2^, Xiaokun Li^1^,Yi Wang^1^, Jingying Wang^1^,*, Guang Liang^1^,*

*^1^ Chemical Biology Research Center, School of Pharmaceutical Science, Wenzhou Medical University, Wenzhou, Zhejiang, China;*

*^2^ Department of Cardiology, Wenzhou Central Hospital, Wenzhou, 325300, Zhejiang, China;*

**Supplementary Tables and Figures**

**Table S1.** Primers used in real-time qPCR assay.

**Figure S1.** APOE^-/-^ mice were fed with HFD for 8 weeks, and then treated with AG1478 (AG, 10mg/kg) or 542 (10 mg/kg) for 8 weeks by oral gavage. (A-C) Serum total cholesterol (TCH), Low-density lipoprotein (LDL) and total triglyceride (TG) were detected as described in the methods (n = 7 in each group; ***p*<0.01 vs. APOE -HFD alone).

**Figure S2.** C57BL/6 WT mice were fed with High Fatty Diet for 8 weeks, and then treated with AG1478 (AG, 10mg/kg) or 542 (10 mg/kg) for 8 weeks by oral gavage. Serum LDL (A) and TG (B) were detected as described in the methods (**p*<0.05, ***p*<0.01 vs. Con). (C) Mouse body weight (n = 7 in each group; ***p*<0.01 vs. HFD alone, ****p*<0.001 vs. Con).

**Figure S3.** Post-treatment of EGFR inhibitors attenuated PA-induced TNF-α and ANP expression in H9c2 cells.

**Figure S4.** Gels/blots with the cropping lines.

**Supplemental Tables and Figures**

**Table S1.** Primer sequences for real-time quantitative PCR

| **Gene** | **species** | **FW** | **RW** |
| --- | --- | --- | --- |
| TNF-α | rat | TACTCCCAGGTTCTCTTCAAGG | GGAGGCTGACTTTCTCCTGGTA |
| ICAM-1 | rat | AGATCATACGGGTTTGGGCTTC | TATGACTCGTGAAAGAAATCAGCTC |
| MCP-1 | rat | GTCACCAAGCTCAAGAGAGAGA | GAGTGGATGCATTAGCTTCAGA |
| IL-6 | rat | GAGTTGTGCAATGGCAATTC | ACTCCAGAAGACCAGAGCAG |
| ANP | rat | GAGGAGAAGATGCCGGTAG | TCAGAGAGGGAGCTAAGTG |
| TGF-β | rat | GCAACAACGCAATCTATGAC | CCTGTATTCCGTCTCCTT |
| β-Actin | rat | AAGTCCCTCACCCTCCCAAAAG | AAGCAATGCTGTCACCTTCCC |
| BNP | mouse | GTCAGTCGTTTGGGCTGTAAC | AGACCCAGGCAGAGTCAGAA |
| TGF-β | mouse | TGACGTCACTGGAGTTGTACGG | GGTTCATGTCATGGATGGTGC |
| Collagen1 | mouse | TGGCCTTGGAGGAAACTTTG | CTTGGAAACCTTGTGGACCAG |
| C-TGF | mouse | ACTATGATGCGAGCCAACTGC | TGTCCGGATGCACTTTTTGC |
| IL-6 | mouse | GAGGATACCACTCCCAACAGACC | AAGTGCATCATCGTTGTTCATACA |
| TNF-α | mouse | TGATCCGCGACGTGGAA | ACCGCCTGGAGTTCTGGAA |
| VCAM-1 | mouse | TGCCGAGCTAAATTACACATTG | CCTTGTGGAGGGATGTACAGA |
| β-Actin | mouse | CCGTGAAAAGATGACCCAGA | TACGACCAGAGGCATACAG |

**Figure S1.** APOE^-/-^ mice were fed with HFD for 8 weeks, and then treated with AG1478 (AG, 10mg/kg) or 542 (10 mg/kg) for 8 weeks by oral gavage. (A-C) Serum total cholesterol (TCH), Low-density lipoprotein (LDL) and total triglyceride (TG) were detected as described in the methods (n = 7 in each group; ***p*<0.01 vs. APOE -HFD alone).

**Figure S2.** C57BL/6 WT mice were fed with High Fatty Diet for 8 weeks, and then treated with AG1478 (AG, 10mg/kg) or 542 (10 mg/kg) for 8 weeks by oral gavage. Serum LDL (A) and TG (B) were detected as described in the methods (**p*<0.05, ***p*<0.01 vs. Con). (C) Mouse body weight (n = 7 in each group; ***p*<0.01 vs. HFD alone, ****p*<0.001 vs. Con).

**Figure S3. Post-treatment of EGFR inhibitors attenuated PA-induced TNF-α and ANP expression in H9c2 cells.** H9c2 were incubated with PA (Palmitate, 100μM) for 6h. At the first hour after PA incubation, AG1478 (AG, 10μM) or 542 (2.5, 5, 10μM) were added into the cell cultural medium for 5h. The mRNA levels of VCAM-1 (A) and ICAM-1 (B) were detected by q-PCR and normalized by β-actin. Bars represent the mean±SD of 3 independent experiments.

**Figure S4.** Gels/blots with the cropping lines.
